# Risk of colorectal cancer by socioeconomic position and history of mental illness – a national nested case-control study

**DOI:** 10.1186/s12885-026-16093-0

**Published:** 2026-04-25

**Authors:** Erik Osterman, Elisavet Syriopoulou, Anna Martling, Therese M-L Andersson, Caroline Nordenvall

**Affiliations:** 1 Department of Molecular Medicine and Surgery, Karolinska Institute, Stockholm, Sweden; 2 Department of Surgical Sciences, Uppsala University, Uppsala, Sweden; 3 Department of Medical Epidemiology and Biostatistics, Karolinska Institute, Stockholm, Sweden; 4 Department of Pelvic Cancer, Colorectal Surgery Unit, Karolinska University Hospital, Stockholm, Sweden

**Keywords:** Colorectal cancer, Socioeconomic position, Incidence risk ratio, Mental illness, Antidepressants

## Abstract

**Aim:**

Whether socioeconomic factors are associated with colorectal cancer (CRC) risk remains unknown, with inconsistent results from different countries. The hypothesis was that both socioeconomic factors and the presence of psychiatric comorbidities are risk factors for CRC.

**Method:**

CRCBaSe, a registry linkage project, was used for a nested case‒control study with 78,043 CRC patients and 431,105 controls matched by age, sex, and county of residence. Socioeconomic factors (income, education, civil status, and birth country) and history of mental illness were used to obtain incidence rate ratios (hazard ratios).

**Results:**

Patients in the middle-income quartile had an increased rate of CRC compared with those in the lowest-income quartile (HR 1.05, 95%CI 1.03–1.08 for Q2 vs. Q1, and HR 1.04 95%CI 1.021.07 for Q3 vs. Q1), whereas there was no association with high income or educational level. Compared with those born in Sweden, those born in other Nordic countries had an increased rate of CRC (HR 1.06 95%CI 1.02–1.10), and those born outside of the EU had a decreased rate (HR 0.80 95%CI 0.77–0.83). A history of mental illness was associated with the rate of CRC even after adjusting for socioeconomic factors: mild mental illness, HR 0.81 (95%CI 0.78–0.83); and severe mental illness, HR 1.85 (95%CI 1.74–1.96).

**Conclusion:**

These findings confirm that the effect of socioeconomic position on CRC risk is heterogeneous and small in Sweden and that severe mental illness is a separate risk factor. Further investigations into the mechanisms that drive the increased risk of CRC in patients with mental illness are warranted.

**Supplementary Information:**

The online version contains supplementary material available at 10.1186/s12885-026-16093-0.

## Background

The incidence of colorectal cancer (CRC) is increasing worldwide, especially among young people, and has been attributed in part to lifestyle factors [1]. Diets rich in fat and meat and low in greens and dietary fibre increase the risk of CRC. Additionally, low physical activity, obesity, cigarette smoking and high alcohol consumption further increase the risk [1]. 

Socioeconomic position (SEP), such as educational level, income, and origin, is associated with different health behaviours. Smoking and poor dietary patterns are associated with lower SEP, and lower educational levels are associated with more physically demanding employment [2–5]. Low SEP is correlated with refraining from seeking care [6] and lower health literacy, which can impact health-related behaviours [7]; thus, SEP can be considered a proxy for health engagement [5]. Participation in CRC screening is lower among the most deprived, potentially affecting the incidence, stage at diagnosis and ultimately survival of patients with CRC [8]. Variations in survival by SEP, age and sex have been observed in many cancers [9–11].

In the US, a lower SEP is associated with an increased risk of CRC [12]. In Europe, with various healthcare systems, the evidence is heterogeneous. Older studies in Finland, Italy and Sweden have shown an opposite trend to that in the US, i.e., higher SEP is correlated with an increased incidence of CRC [11, 13, 14]. This finding was not replicated in a more recent Swedish study that included cases up to 2010 [15]. Data from Denmark are in line with American data, where low SEP is a risk factor for CRC [16]. The country of birth, although not always linked to SEP, has been associated with the risk of CRC, which is usually lower in immigrants, the healthy migrant paradox [17, 18]. The significance of SEP may change over time due to shifts in demographics, policy decisions and interventions, highlighting the need for periodic evaluation.

Persons with severe psychiatric diseases (bipolar, depressive disorders and psychotic disease) are often socioeconomically deprived and have worse all-cause survival [19, 20]. The incidence of several cancers, including CRC, has been reported to be lower in people with schizophrenia [21], but other studies suggest the opposite [22]. Persons with depression and anxiety disorders treated with antidepressants have been found to have a decreased risk of CRC [23]. As with SEP, the explanation for these differences is probably multifactorial, including underlying biological factors, disparities in access to care, health behaviour, exposure to certain medications and variations in data quality.

### Aim

This study aimed to examine the risk of CRC across different SEP groups in relation to a history of mental illness. The hypothesis was that persons with low SEP, mental illness or both have a greater risk of CRC and that the elevated risk among those with mental illness is mediated by educational level and income.

## Method

The study was approved by the Regional Board of the Ethical Committee in Stockholm (DNR: 2014/71–31, 2018/328–32) and by the National Ethical Committee (DNR: 2021–00342). The manuscript was prepared according to the Strengthening the Reporting of Observational Studies in Epidemiology (STROBE) guidelines for case‒control studies [24].

### Patient data

Patient data originated from the Colorectal Cancer Database (CRCBaSe), a register-linkage of the Swedish Colorectal Cancer Registry (SCRCR) and national registries at the National Board of Welfare and Statistics Sweden described in more detail elsewhere [25]. The SCRCR is funded jointly by the healthcare regions of Sweden for quality assurance and research. It covers all Swedish colorectal cancers since 2007 with data on treatment, staging and follow-up. Registry data were linked via Swedish personal identification numbers, which are unique numbers issued to everyone living in Sweden.

CRCBaSe includes controls, allowing for nested case‒control studies of the risk of CRC. All cases are matched with six controls from the general population in CRCBaSe based on the year of birth, sex, county of residence and being free of CRC on the matching date. Patients can also serve as controls before their CRC diagnosis. This study included all adults (≥ 18 years old) with a first-time diagnosis of CRC in Sweden between 2010 and 2021 along with their matched controls.

### Exposures

SEP was defined on the basis of four indicators: (i) individual part of disposable household income, (ii) highest education level achieved, (iii) civil status and (iv) birth country. Statistics Sweden collects these variables annually from government agencies. The average individual share of disposable household income two years before diagnosis/matching was divided into quartiles to categorise individuals into high- and low-income groups. The quartiles (Q) were created separately for individuals above and below 65 years of age of the same sex, with income quartile 1 (Q1) being the most deprived and income quartile 4 (Q4) being the least deprived. Education level at diagnosis was categorised as < 9 years, 9–12 years and > 12 years. Civil status at diagnosis was categorised into two groups (living alone or not) on the basis of information on registered marriages, divorces, partnerships, and cohabitation, along with individual and household income data. Patients with an individual income-to-weighted household income ratio smaller than 0.9 or larger than 1.1 were reclassified as living with someone, even if they were not married or in a registered partnership. The birth country was categorised as Swedish-born, Nordic-born (outside Sweden), EU-born (outside the Nordics), or non-EU-born.

A history of mental illness was identified from the patient registry covering psychiatric inpatient care since 1974 (complete coverage since 1987), psychiatric outpatient care since 2001, and the national prescription registry containing all prescriptions in Sweden since July 2005. Both registries are government-run and mandatory to report to for health care providers and pharmacies. Healthcare contacts 5 years before the cancer diagnosis, International Classification of Disease 10 code (ICD10 used since 1997) of F20- F22, and F30-F39, which are the Swedish ICD10 codes for schizophrenic disorders, psychosis, bipolar disorders and depressive disorders, were used to identify patients with a history of mental illness before CRC diagnosis. Prescriptions of medications 5 years prior to CRC diagnosis with Anatomical Therapeutic Chemical Classification System (ATC) [26] codes N06A, N06B, and N06C, which are antidepressants, central stimulants and combinations, were used to identify patients with a history of mental illness who did not have inpatient or outpatient contact with specialised psychiatric care. Patients with a diagnosis in the patient registry were classified as having a history of severe mental illness, as they required specialised care. Those on an antidepressant prescription with at least 2 prescriptions, multiple antidepressants or prescriptions of 90 or more defined daily doses, e.g., at least 3 months of use [26], and no psychiatric diagnoses in the patient registry, or on a central stimulant, were classified as having a history of mild mental illness. Patients without codes or prescriptions were classified as having no history of mental illness. Separate variables for antidepressant medications with different chemical structures and mechanisms of action were created: nonselective monoamine reuptake inhibitors and monoamine inhibitors (MAO), selective serotonin reuptake inhibitors (SSRI), serotonin and norepinephrine reuptake inhibitors (SNRI), tetracyclic antidepressants (TCA), lithium, other antidepressive drugs, and antipsychotic drugs. In addition, the prescribed defined daily doses were extracted for the MAO, SSRI, SNRI and TCA drugs from the national prescribed drug register.

### Covariates and strata

Other variables included in the dataset were sex (male, female), age at diagnosis (years) and county of residence at matching to control for matching sourced from Statistics Sweden. The location of the cancer (colon or rectum) was sourced from the SCRCR to facilitate separate analysis for each location.

### Statistical analysis

A complete case analysis was used since there were no cases with missing data on matching variables and only a few cases with missing data for the exposures and outcomes.

Conditional logistic regression was used to calculate the incidence rate ratio over time, i.e., hazard ratios (HRs), with 95% confidence intervals (95% CIs), for CRC patients, including all individuals [27]. Additional conditional logistic regression models were fitted in four subsets of the population, one for each location (colon cancer and rectal cancer) and one for each sex (males and females).

For all CRC patients as well as for each of the four subsets described above, 8 models were fitted, each including one of the exposure sets described below:

i) Individual part of disposable household income.
ii) Highest education achieved.
iii) Birth country.
iv) Civil status.
v) All SEP indicators listed above (i-iv).
vi) Income (i) and education (ii) and the interaction between them.
vii) History of mental illness (severe, mild, or no mental illness).
viii) All SEP indicators listed above (i-iv) and history of mental illness (vii).

For the exposure set (vi), we tested the significance of including an interaction. The interaction was not statistically significant in any of the subsets, and the results from (vi) are therefore not presented (likelihood ratio test p 0.1). In addition, to assess the impact of specific characteristics and medications on mental illness groups, since the effects of severe mood disorders and psychotic diseases are not necessarily homogenous, an analysis of the rate of CRC caused by prescribed medications was performed. Nine models were fitted for any antidepressant, category of prescribed medication (MAO, SSRI, SNRI, TCA, other antidepressants, antipsychotics and lithium) together, or separately (7 models). These models were adjusted for mental illness (severe, mild, none) and all SEP indicators. More detailed categories to define the severe mental illness group, i.e., severe depression, schizophrenic/psychotic disorders and bipolar disorder, were used to fit two models with and without prescribed medication categories. In addition, the dose‒response of MAOs, SSRIs, SNRIs and TCAs and the rate of CRC were investigated by including each of the four prescription types with the prescribed defined daily doses (0, 1–89, 90–179 and ≥ 180 defined daily doses) in the model in addition to mental illness (no, mild, severe) and all SEP indicators. The groups correspond to the four common groups of antidepressants, and the daily doses were selected to correspond to 3, 6 and > 6 months of treatment at the effective dose in line with the available packages. The total number of models fitted was 55.

All analyses were performed via R statistical software (v4.1.2; R Core Team 2021) [28]. The function clogit() from the package *survival* was used to calculate the conditional logistic regressions [29]. The HRs from models i-iii and vii-viii were visualised via forest plots to facilitate the comparison of results between subsets.

## Results

There were 78,043 cases and 431,105 controls in the CRCBaSe cohort. The median age was 73 years, and 53% of the cases and controls were men. Cases were more likely to be in income groups Q2 and Q3, to have shorter education and to be Swedish born (Table 1). A history of mental illness was more common among the patients (Table 1).

**Table 1:** Demographics of patients with colorectal cancer in Sweden from 2010-2021 and controls from CRCBaSe

		Total	Control	Case
Characteristic	Categories	N (%)	N (%)	N (%)
Sex	***Male***	269,489 (52.9%)	227,912 (52.9%)	41,577 (53.3%)
	***Female***	239,659 (47.1%)	203,193 (47.1%)	36,466 (46.7%)
Age (years)	***Median (IQR)***	73.0 (65.0 - 80.0)	73.0 (65.0 - 80.0)	73.0 (65.0 - 80.0)
Income Quartile	***Q1***	115,971 (22.8%)	98,381 (22.8%)	17,590 (22.5%)
	***Q2***	127,120 (25.0%)	107,036 (24.8%)	20,084 (25.7%)
	***Q3***	130,056 (25.5%)	109,753 (25.5%)	20,303 (26.0%)
	***Q4***	133,078 (26.1%)	113,377 (26.3%)	19,701 (25.2%)
	***Missing***	2,923 (0.6%)	2,558 (0.6%)	365 (0.5%)
Education	***-9y***	127,127 (25.0%)	107,194 (24.9%)	19,933 (25.5%)
	***9y-12y***	187,727 (36.9%)	158,527 (36.8%)	29,200 (37.4%)
	***12y-***	187,186 (36.8%)	159,210 (36.9%)	27,976 (35.8%)
	***Missing***	7,108 (1.4%)	6,174 (1.4%)	934 (1.2%)
Birth country	***Sweden***	441,420 (86.7%)	373,315 (86.6%)	68,105 (87.3%)
	***Nordic***	23,194 (4.6%)	19,426 (4.5%)	3,768 (4.8%)
	***EU***	16,493 (3.2%)	13,851 (3.2%)	2,642 (3.4%)
	***Non-EU***	28,041 (5.5%)	24,513 (5.7%)	3,528 (4.5%)
Civil status	***Alone***	203,055 (39.9%)	171,897 (39.9%)	31,158 (39.9%)
	***Not alone***	306,093 (60.1%)	259,208 (60.1%)	46,885 (60.1%)
History of mental illness	***No***	441,125 (86.6%)	372,953 (86.5%)	68,172 (87.4%)
	***Mild***	61,809 (12.1%)	53,533 (12.4%)	8,276 (10.6%)
	***Severe***	6,214 (1.2%)	4,619 (1.1%)	1,595 (2.0%)
Cancer location	***Colon***	53,211 (10.5%)	0 (0.0%)	53,211 (68.2%)
	***Rectum***	24,832 (4.9%)	0 (0.0%)	24,832 (31.8%)
	***None***	431,105 (84.7%)	431,105 (100.0%)	0 (0.0%)

*IQR * Interquartile range, *Y * Years

Q: Quartile’, Q1 and Q4 refer to the lowest and highest income quartiles, respectively

### Income and education

**Fig. 1:**
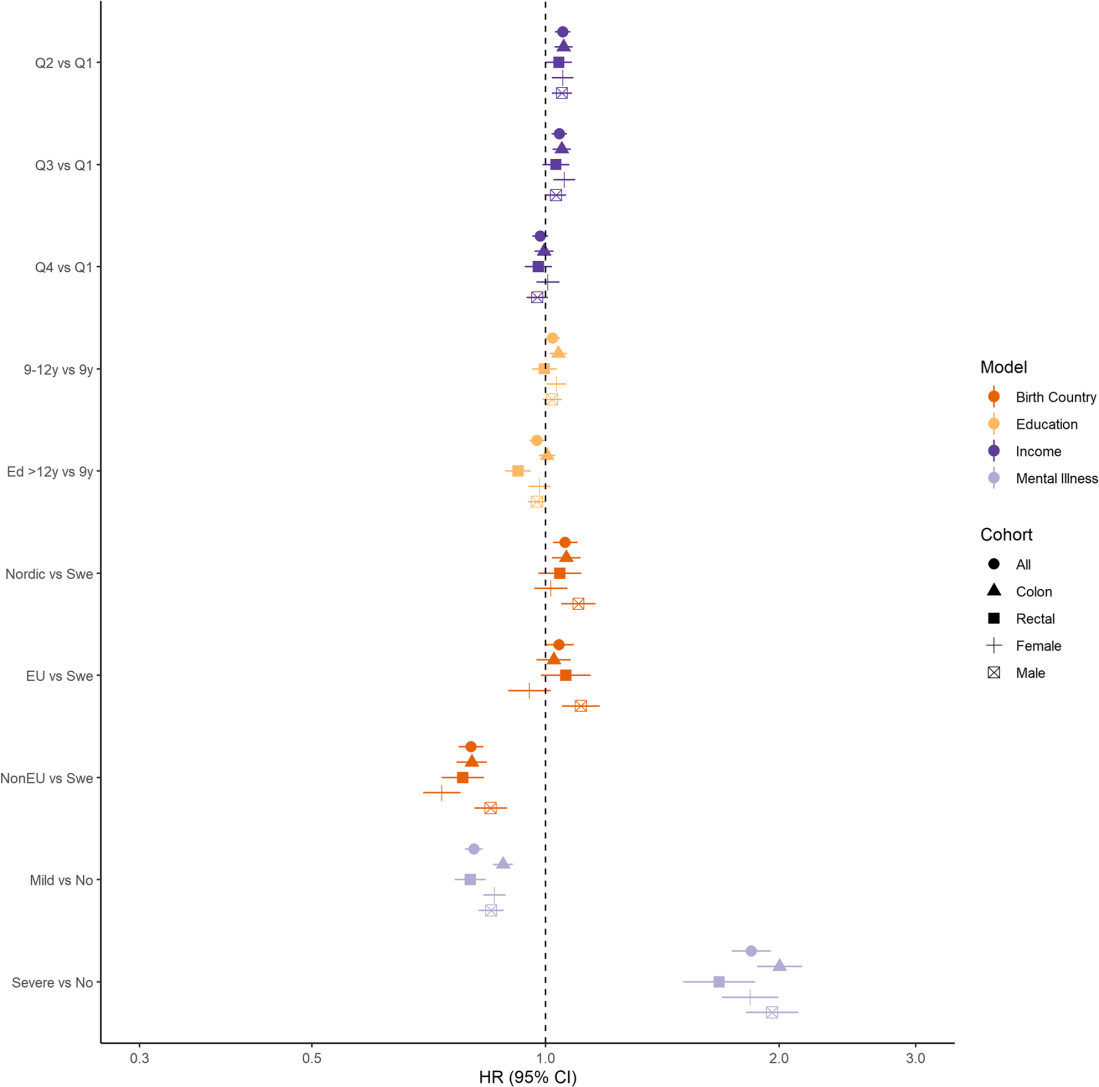
Forest plot for the risk of colorectal cancer, colon and rectal cancer and colorectal cancer by sex and separately for each SEP and mental illness indicator. Hazard ratios were obtained from conditional logistic regression models including one indicator at a time, with bars representing 95% confidence intervals. Matching by sex, age, and county of residence

The rate of CRC was higher in income Q2 and Q3 than in Q1 (Q2 HR 1.05, 95% CI 1.03–1.08 and Q3 HR 1.04 95% CI 1.02–1.07), whereas no such association was present for income Q4, as presented in both Table 2; Fig. 1. Analysis by tumour location revealed similar estimates. The effect of income was similar in males and females.

**Table 2:** Hazard ratio of colorectal cancer according to SEP indicators and history of mental illness in Sweden from 2010-2021 calculated using controls from CRCBaSe

	Indicator	CRC	Colon	Rectal	Female	Male
Exposure	Level	*HR (95% CI)*	*HR (95% CI)*	*HR (95% CI)*	*HR (95% CI)*	*HR (95% CI)*
Income	Q2 vs Q1	1.05 (1.03-1.08)	1.05 (1.03-1.08)	1.04 (1.00-1.08)	1.05 (1.02-1.09)	1.05 (1.02-1.08)
	Q3 vs Q1	1.04 (1.02-1.07)	1.05 (1.02-1.08)	1.03 (0.99-1.07)	1.06 (1.02-1.09)	1.03 (1.00-1.06)
	Q4 vs Q1	0.98 (0.96-1.01)	1.00 (0.97-1.02)	0.98 (0.94-1.02)	1.01 (0.97-1.04)	0.98 (0.95-1.01)
Education	9-12y vs <9y	1.02 (1.00-1.04)	1.04 (1.01-1.06)	1.00 (0.96-1.03)	1.03 (1.00-1.06)	1.02 (0.99-1.05)
	>12y vs <9y	0.97 (0.95-1.00)	1.00 (0.98-1.03)	0.92 (0.89-0.96)	0.98 (0.95-1.01)	0.97 (0.95-1.00)
Birth country	Nordic vs Sweden	1.06 (1.02-1.10)	1.06 (1.02-1.11)	1.04 (0.98-1.11)	1.02 (0.97-1.07)	1.1 (1.05-1.16)
	EU vs Sweden	1.04 (1.00-1.09)	1.02 (0.97-1.08)	1.06 (0.99-1.14)	0.95 (0.9-1.02)	1.11 (1.05-1.17)
	Non-EU vs Sweden	0.80 (0.77-0.83)	0.80 (0.77-0.84)	0.78 (0.73-0.83)	0.74 (0.70-0.78)	0.85 (0.81-0.89)
Civil status	Partner vs not	1.00 (0.98-1.01)	1.00 (0.98-1.02)	1.03 (1.00-1.06)	1.01 (0.99-1.03)	1.01 (0.98-1.03)
Mental illness	Mild vs no	0.81 (0.79-0.83)	0.88 (0.86-0.91)	0.80 (0.76-0.84)	0.86 (0.83-0.89)	0.85 (0.82-0.88)
	Severe vs no	1.84 (1.74-1.95)	2.00 (1.87-2.14)	1.67 (1.51-1.86)	1.83 (1.69-1.99)	1.96 (1.81-2.12)

Each SEP was entered separately. Sex-, age-, and county of residence matched controls. Conditional logistic regression

95% CI: 95% confidence interval

*EU* Non-Nordic EU countries,  *HR* Hazard ratio, *Q * quartile, *SEP* Socioeconomic position, *Y* Years of education

Educational level was not associated with overall CRC rates. For rectal cancer, there was a decreased rate for those with > 12 years of education (HR 0.91 95% CI 0.88–0.94) (Table 2). There was an increased incidence of colon cancer in the middle-educated group. The rate of CRC was greater in females with 9–12 years of education than in females with less than 9 years of education (HR 1.03 95% CI 1.00–1.06), but no such increase was observed for females with > 12 years of education (Table 2). Similar estimates to those for females were observed for males, although the difference was not statistically significant.

### Birth country and civil status

Compared with those born in Sweden, those born in the Nordics or EU had an increased rate of CRC (Nordic HR 1.06 95% CI 1.02–1.10 and EU HR 1.04 95% CI 1.00-1.09), whereas those born outside the EU had a decreased rate (HR 0.80 95% CI 0.77–0.83) (Table 2). Analysis by tumour location yielded similar results, but the results differed between the sexes. In females, there was a small increase in the CRC rate for those who were Nordic-born compared with Sweden-born individuals and a small decrease for those who were EU-born, although the difference was not statistically significant. The percentage of non-EU-born females was lower (HR 0.74 95% CI 0.70–0.78). For males, the rate of CRC was increased if they were born in the Nordics or EU (HR for EU 1.11 95% CI 1.05–1.17); however, being born outside of the EU was associated with a lower rate of CRC (HR 0.85 95% CI 0.81–0.89) (Table 2). Civil status did not impact the rate of CRC. There was no difference in the effect of civil status between the sexes. Entering all SEP indicators together into the model did not change the estimates for CRC (Supplementary Table 1).

### History of mental illness

**Fig. 2:**
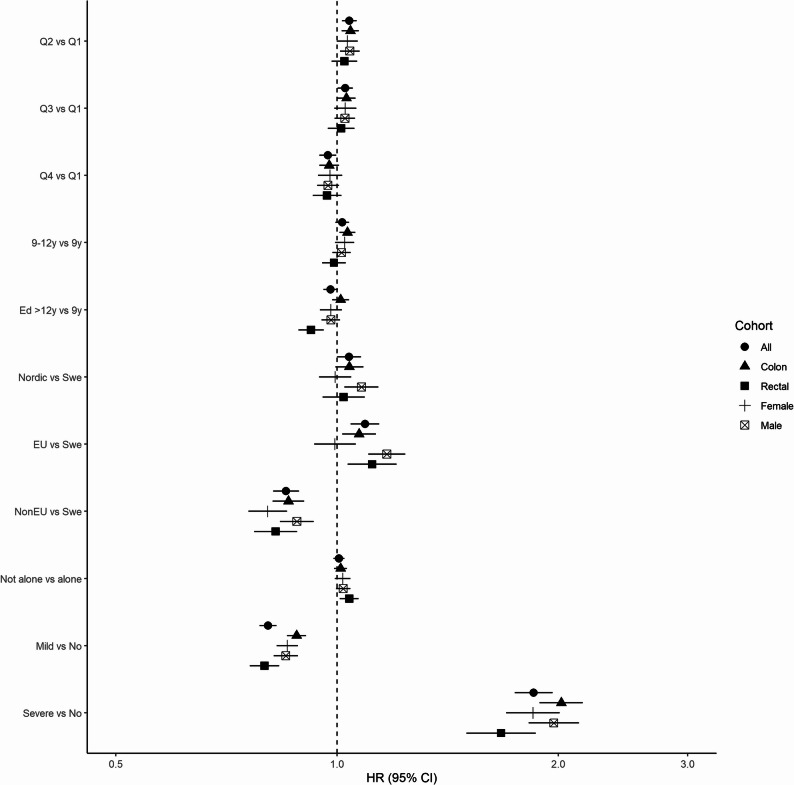
Forest plot for the risk of colorectal cancer, colon and rectal cancer and colorectal cancer by sex obtained from a model including a history of mental illness (MI) and all SEP indicators. Hazard ratios were obtained from a conditional logistic regression model with bars representing 95% confidence intervals. Matching by sex, age, and county of residence

The rate of CRC was decreased in those with mild mental illness (HR 0.81, 95% CI 0.79–0.83), as presented in Table 2; Fig. 1. Adjusting the model for all SEP indicators yielded almost identical estimates (Fig. 2 and Supplementary Table 2). Patients with severe mental illness had an increased rate of CRC (HR 1.84, 95% CI 1.74–1.95), and adjustment for SEP yielded similar estimates (HR 1.85, 95% CI 1.74–1.96). The same patterns were observed after stratification by tumour location and sex (Supplementary Table 2).

### Prescribed drugs

The most prescribed antidepressants were SSRIs, with 45,806 (9.0%) of patients and controls having at least two prescriptions or over 90 defined daily doses (Supplementary Table 3). When the model was adjusted for mental illness, all SEP indicators and one prescribed medication at a time, the effect of mild mental illness was similar to that of the models without adjustment for the prescribed medication (Supplementary Tables 4 and Supplementary Fig. 1). The prescription of any antidepressants reduced the rate of CRC (HR 0.78, 95% CI 0.71–0.85), as did SSRIs (HR 0.94, 95% CI 0.89–0.99) and TCAs (HR 0.87, 95%CI0.83-0.92). Lithium, which is commonly prescribed for bipolar disorders, was associated with a lower rate of CRC (HR 0.74, 95%CI 0.63–0.87). When all the antidepressant medications were added to the model at once (Table 3 and Supplementary Fig. 1), the effects of the SSRIs and TCAs remained, while the effect of lithium was reversed. SNRIs were associated with an increased risk of CRC in the model with all antidepressants (Table 3).

**Table 3:** Hazard ratio of colorectal cancer obtained from three models adjusted with all SEP indicators and then including either the crude (Model 3) or detailed definitions of the history of mental illness (Model 1 and Model 2) and prescribed drugs (Model 2 and Model 3)

	Indicator	Model 1: Detailed mental illness definition	Model 2: Adjusting for prescribed drugs	Model 3: Detailed mental illness definition and adjusting for prescribed drugs
Exposure	Level	*HR (95% CI)*	*HR (95% CI)*	*HR (95% CI)*
Mental illness group	Mild	0.80 (0.78-0.82)	0.94 (0.89-1.00)	0.96 (0.91-1.02)
	Severe		2.93 (2.73-3.15)	
	Severe depression	1.66 (1.02-2.69)		2.18 (1.34-3.56)
	Psychosis	1.13 (1.04-1.23)		1.89 (1.70-2.11)
	Bipolar	3.57 (3.27-3.90)		4.09 (3.74-4.48)
Income	Q2 vs Q1	1.03 (1.01-1.06)	1.03 (1.01-1.06)	1.03 (1.01-1.05)
	Q3 vs Q1	1.02 (1.00-1.05)	1.02 (0.99-1.04)	1.02 (0.99-1.04)
	Q4 vs Q1	0.97 (0.94-0.99)	0.96 (0.94-0.99)	0.96 (0.94-0.99)
Education	9-12y vs <9y	1.01 (0.99-1.04)	1.01 (0.99-1.04)	1.01 (0.99-1.04)
	>12y vs <9y	0.98 (0.96-1.00)	0.98 (0.96-1.00)	0.98 (0.95-1.00)
Birth country	Nordic vs Sweden	1.04 (1.00-1.08)	1.04 (1.00-1.08)	1.04 (1.00-1.08)
	EU vs Sweden	1.09 (1.05-1.14)	1.09 (1.04-1.14)	1.09 (1.04-1.14)
	Non-EU vs Sweden	0.85 (0.82-0.89)	0.85 (0.82-0.89)	0.85 (0.82-0.89)
Civil status	Partner vs not	1.00 (0.99-1.02)	1.00 (0.98-1.02)	1.00 (0.98-1.02)
Medication	MAO		0.90 (0.84-0.96)	0.88 (0.83-0.95)
	SSRI		0.91 (0.86-0.96)	0.88 (0.83-0.93)
	SNRI		1.10 (1.03-1.18)	1.08 (1.01-1.16)
	TCA		0.91 (0.86-0.96)	0.88 (0.84-0.93)
	Other antidepressant		1.09 (0.97-1.23)	1.07 (0.95-1.21)
	Lithium		1.28 (1.08-1.52)	1.08 (0.91-1.29)
	Antipsychotics		0.43 (0.40-0.46)	0.53 (0.49-0.58)

Each column represents a different model. In the models adjusted for prescriptions, all drugs were entered into the model at once

*MAO* drugs that act on MAO receptors or uptake pumps, *SSRI* selective serotonin reuptake inhibitors, *SNRI* serotonin and norepinephrine reuptake inhibitors, *TCA* atypical tetracyclic antidepressants, * antipsychotics * other antipsychotic medications

The dose‒response effects of antidepressants were investigated by including a categorical variable of the number of defined daily doses of the prescriptions in each of the models and adjusting for SEP and mental illness. Compared with no dose, 1–89 defined daily doses of MAO were associated with a reduced rate of CRC (HR 0.93, 95% CI 0.87–0.99). More than 180 defined daily doses of SSRI (HR 0.93, 95% CI 0.88–0.98) were associated with a reduced rate of CRC. Compared with no dose, ≥ 180 defined daily doses of TCAs were associated with a reduced rate of CRC (HR 0.83, 95% CI 0.81–0.88). SNRIs were not associated with the rate of CRC when analysed by defined daily doses.

### Detailed categories of severe mental illness

There were 2,217 (0.4%) patients and controls with bipolar disorder and 3,905 (0.8%) with schizophrenia and/or psychosis. When severe mental illness was modelled in detail, severe depression had a significant effect on the rate of CRC, whereas psychosis had a lesser effect while bipolar disorders led to a significantly greater CRC rate. When the model was adjusted for specific mental illness groups and medications, MAOs, SSRIs, TCAs and antipsychotics were associated with a protective effect, whereas severe mental illnesses remained associated with an increased rate of CRC (Table 3).

## Discussion

In this case‒control study of more than 70,000 incident CRC cases and 400,000 controls, we explored how the effect of SEP and history of mental illness on CRC risk. Mild mental illness was linked to a decreased risk of CRC and severe mental illness was linked to an increased risk of CRC, effects that remained even after adjusting for SEP. Reagrding the associations between SEP and CRC risk, income, education and civil status had a small or no effect, while the risk by country of origin varied.

The impact of SEP and mental illness is continually influenced by evolving health awareness and preventive measures for CRC. To enhance our understanding of the roles of SEP and mental illness, a modern, nationwide study spanning 11 years and incorporating detailed data on socioeconomic position, psychiatric diagnoses, and medication use was conducted. The SCRCR provides comprehensive coverage of CRC cases in Sweden [25]. By including six controls per case, a high relative efficiency was achieved in the conditional logistic regression models. This study is unique in its capacity to robustly examine the influence of both SEP and mental illness. No estimates for the effects of age, sex, county of residence or calendar year can be made, as these were the factors used for matching. Since both cases and controls are obtained from Swedish registers and the impact of SEP can vary across countries, caution should be exercised when generalising these findings to other populations. The missing data in this setting would be challenging to impute, e.g., imputing the education status of individuals with missing data based on the observed data requires strong assumptions.

Other limitations include a lack of information on mediating factors such as smoking, diet and lifestyle habits. As there is a correlation between SEP and mental illness and other risk factors, there is a risk of residual confounding in the results. However, estimating the total effect of mental illness requires adjusting for SEP but not BMI or smoking if these are thought of as mediators of the effect of mental illness or SEP.

This study suggests that an increasing level of income does not impact the risk of CRC (as Q4 was not associated with the rate of CRC). The increase in risk for middle-income persons was small and likely from lifestyle differences compared to individuals with Q1 and Q4 incomes [31, 32].In the analyses of educational level, there was only one observed association, where a higher educational level was associated with a lower risk of rectal cancer. This association may be explained in part by smoking, a known risk factor for rectal cancer, which is less common in more educated populations [30]. Increasing data support that colon cancer and rectal cancer are two unique diseases with different patterns of risk factors; therefore, analyses were also stratified by location. Additionally, higher educational attainment is often associated with greater health awareness, leading individuals to adopt healthier lifestyles and to be more proactive in seeking medical care [31, 32]. The effect of SEP was small, and the effect direction depended on the location of the tumour. This study is unique in that it is based on information at the individual level, whereas previous studies have used primarily area-level indicators or are based on older data [11–16]. The observed magnitude in the present study is in agreement with the previously mentioned effects.

This study reaffirmed the significant role of birth country in CRC risk. Immigrants from neighbouring countries showed an increased risk of CRC, in line with previous studies reporting higher CRC incidences in Norway and Denmark, whereas Finland and Iceland have rates similar to those of Sweden [33]. This may explain the elevated CRC risk in Nordic immigrants, despite Finland being the most common Nordic country of origin for immigration [34]. Similarly, Sweden’s lower CRC incidence than the EU average may account for the increased risk observed in persons born in other EU countries [33]. However, immigrants from non-EU countries presented a lower CRC risk, which is consistent with findings from other studies [17, 18].

The findings of the present study support previous findings that the use of some antidepressant medications may reduce CRC risk [23]. A prior hospital-based study revealed that SSRI medication reduced the risk of colon cancer by more than 50% and that of rectal cancer by nearly 30% [35]. In contrast, a Danish population-based study of 10,000 cases revealed no protective effect of antidepressants [36], which was also in agreement with a study from the Women’s Health Initiative [37]. Here, a history of mild mental illness was associated with an decreased risk, potentially mediated by some but not all antidepressants, suggesting different mechanisms. There may be biological mechanisms explaining why SSRIs could offer protective benefits, whereas SNRIs do not. Alternatively, the associations could reflect a specific group of persons who have regular healthcare visits and greater health awareness than those who have mental illness not treated with medication.

The evidence for severe mental illness is conflicting with reports of decreased risk [21], and increased risks of colorectal cancer in other reports [22]. Interestingly, this association remained after adjusting for differences in SEP, contrary to our hypothesis. The high prevalence of smoking and other lifestyle factors could explain some of the increased risk associated with severe mental illness [38, 39]. However, previous studies have also reported that the prescription of antipsychotic medicines increases the CRC risk in patients with schizophrenia. Severe mental illness impacts participation in screening, which could impact the stage at diagnosis but also increase the incidence when precursor lesions are not removed [40]. There is evidence from ex vivo studies and mice that some antipsychotics may have anticancer effects [41]. In this study, antipsychotics, but not lithium, were associated with a lower risk of CRC.

### Future perspectives

Understanding the factors contributing to health inequalities is crucial for developing targeted interventions to improve health outcomes. As the influence of SEP changes over time, regular evaluations are essential. Moreover, further research into the effects of medications on CRC risk and the possible mechanisms behind these associations may enhance the understanding of CRC development.

## Conclusion

There was no increase in the relative risk of clinical significance for income. A high educational level was associated with a decreased rectal cancer risk, whereas country of birth affected CRC risk in different directions. A history of severe mental illness was associated with an increased risk of CRC, and this risk increase remained after adjusting for SEP. These findings suggest that the effect of SEP is heterogeneous in Sweden and more similar to findings from Finland and Italy than Denmark. Further investigations into the mechanisms that drive the associations between severe mental illness, antidepressant drugs and CRC risk are warranted.

## Supplementary Information

Supplementary Material 1.

## Data Availability

The data that support the findings of this study are available from the Swedish Colorectal Cancer Registry, Swedish National Board of Welfare, and Statistics Sweden. Restrictions apply to the availability of these data, which were used under licence for this study. The code used to analyse the data is available upon reasonable request.

## Abbreviations

CI: Confidence Interval
CRC: Colorectal Cancer
EU: Non-nordic European Union countries
HR: Hazard ratio
IQR: Interquartile range
MAO: Non-selective mono amine reuptake inhibitors and mono amine inhibitors
Q1-4: Quartile 1–4
SCRCR: Swedish Colorectal Cancer Registry
SEP: Socioeconomic Position
SNRI: Serotonin and norepinephrine reuptake inhibitors
SSRI: Selective serotonin reuptake inhibitors (
TCA: Tetracyclic antidepressants
Y: Years

## References

[1] Haggar FA, Boushey RP. Colorectal Cancer Epidemiology: Incidence, Mortality, Survival, and Risk Factors. Clin Colon Rectal Surg. 2009;22:191–7. 10.1055/s-0029-1242458. 21037809 10.1055/s-0029-1242458 PMC2796096

[2] Hiscock R, Bauld L, Amos A, Fidler JA, Munafò M. Socioeconomic status and smoking: a review. Ann N Y Acad Sci. 2012;1248:107–23. 10.1111/j.1749-6632.2011.06202.x. 22092035 10.1111/j.1749-6632.2011.06202.x

[3] Backholer K, Spencer E, Gearon E, Magliano DJ, McNaughton SA, Shaw JE, et al. The association between socio-economic position and diet quality in Australian adults. Public Health Nutr. 2016;19:477–85. 10.1017/S1368980015001470. 25989940 10.1017/S1368980015001470 PMC10271031

[4] Gong J, Hutter C, Baron JA, Berndt S, Caan B, Campbell PT, et al. A pooled analysis of smoking and colorectal cancer: timing of exposure and interactions with environmental factors. Cancer Epidemiol Biomark Prev Publ Am Assoc Cancer Res Cosponsored Am Soc Prev Oncol. 2012;21:1974–85. 10.1158/1055-9965.EPI-12-0692. 10.1158/1055-9965.EPI-12-0692 PMC3493822 23001243

[5] Doubeni CA, Major JM, Laiyemo AO, Schootman M, Zauber AG, Hollenbeck AR, et al. Contribution of behavioral risk factors and obesity to socioeconomic differences in colorectal cancer incidence. J Natl Cancer Inst. 2012;104:1353–62. 10.1093/jnci/djs346. 22952311 10.1093/jnci/djs346 PMC3529596

[6] Molarius A, Simonsson B, Lindén-Boström M, Kalander-Blomqvist M, Feldman I, Eriksson HG. Social inequalities in self-reported refraining from health care due to financial reasons in Sweden: health care on equal terms? BMC Health Serv Res. 2014;14:605. 10.1186/s12913-014-0605-2. 25468266 10.1186/s12913-014-0605-2 PMC4254004

[7] Sørensen K, Pelikan JM, Röthlin F, Ganahl K, Slonska Z, Doyle G, et al. Health literacy in Europe: comparative results of the European health literacy survey (HLS-EU). Eur J Public Health. 2015;25:1053–8. 10.1093/eurpub/ckv043. 25843827 10.1093/eurpub/ckv043 PMC4668324

[8] Frederiksen BL, Jørgensen T, Brasso K, Holten I, Osler M. Socioeconomic position and participation in colorectal cancer screening. Br J Cancer. 2010;103:1496–501. 10.1038/sj.bjc.6605962. 20959827 10.1038/sj.bjc.6605962 PMC2990593

[9] Hussain SK, Lenner P, Sundquist J, Hemminki K. Influence of education level on cancer survival in Sweden. Ann Oncol. 2008;19:156–62. 10.1093/annonc/mdm413. 17785761 10.1093/annonc/mdm413

[10] Abdoli G, Bottai M, Moradi T. Cancer Mortality by Country of Birth, Sex, and Socioeconomic Position in Sweden, 1961–2009. PLoS ONE. 2014;9:e93174. 10.1371/journal.pone.0093174. 24682217 10.1371/journal.pone.0093174 PMC3969357

[11] Hemminki K, Li X. Level of education and the risk of cancer in Sweden. Cancer Epidemiol Biomark Prev Publ Am Assoc Cancer Res Cosponsored Am Soc Prev Oncol. 2003;12:796–802. 12917212

[12] Hastert TA, Beresford SAA, Sheppard L, White E. Disparities in cancer incidence and mortality by area-level socioeconomic status: a multilevel analysis. J Epidemiol Community Health. 2015;69:168–76. 10.1136/jech-2014-204417. 25288143 10.1136/jech-2014-204417

[13] Weiderpass E, Pukkala E. Time trends in socioeconomic differences in incidence rates of cancers of gastro-intestinal tract in Finland. BMC Gastroenterol. 2006;6:41. 10.1186/1471-230X-6-41. 17144908 10.1186/1471-230X-6-41 PMC1769383

[14] Tavani A, Fioretti F, Franceschi S, Gallus S, Negri E, Montella M, et al. Education, socioeconomic status and risk of cancer of the colon and rectum. Int J Epidemiol. 1999;28:380–5. 10.1093/ije/28.3.380. 10405837 10.1093/ije/28.3.380

[15] Brooke HL, Talbäck M, Martling A, Feychting M, Ljung R. Socioeconomic position and incidence of colorectal cancer in the Swedish population. Cancer Epidemiol. 2016;40:188–95. 10.1016/j.canep.2016.01.004. 26773279 10.1016/j.canep.2016.01.004

[16] Egeberg R, Halkjær J, Rottmann N, Hansen L, Holten I. Social inequality and incidence of and survival from cancers of the colon and rectum in a population-based study in Denmark, 1994–2003. Eur J Cancer. 2008;44:1978–88. 10.1016/j.ejca.2008.06.020. 18667301 10.1016/j.ejca.2008.06.020

[17] Vanthomme K, Rosskamp M, De Schutter H, Vandenheede H. Colorectal cancer incidence and survival inequalities among labour immigrants in Belgium during 2004–2013. Sci Rep. 2022;12:15727. 10.1038/s41598-022-19322-1. 36130977 10.1038/s41598-022-19322-1 PMC9492689

[18] Shuldiner J, Liu Y, Lofters A. Incidence of breast and colorectal cancer among immigrants in Ontario, Canada: a retrospective cohort study from 2004–2014. BMC Cancer. 2018;18:537. 10.1186/s12885-018-4444-0. 29739346 10.1186/s12885-018-4444-0 PMC5941319

[19] Hudson CG. Socioeconomic Status and Mental Illness: Tests of the Social Causation and Selection Hypotheses. Am J Orthopsychiatry. 2005;75:3–18. 10.1037/0002-9432.75.1.3. 15709846 10.1037/0002-9432.75.1.3

[20] Chan JKN, Correll CU, Wong CSM, Chu RST, Fung VSC, Wong GHS, et al. Life expectancy and years of potential life lost in people with mental disorders: a systematic review and meta-analysis. eClinicalMedicine. 2023;65:102294. 10.1016/j.eclinm.2023.102294. 37965432 10.1016/j.eclinm.2023.102294 PMC10641487

[21] Li H, Li J, Yu X, Zheng H, Sun X, Lu Y, et al. The incidence rate of cancer in patients with schizophrenia: A meta-analysis of cohort studies. Schizophr Res. 2018;195:519–28. 10.1016/j.schres.2017.08.065. 28943096 10.1016/j.schres.2017.08.065

[22] Hippisley-Cox J, Vinogradova Y, Coupland C, Parker C. Risk of Malignancy in Patients With Schizophrenia or Bipolar Disorder: Nested Case-Control Study. Arch Gen Psychiatry. 2007;64:1368. 10.1001/archpsyc.64.12.1368. 18056544 10.1001/archpsyc.64.12.1368

[23] Chubak J, Boudreau DM, Rulyak SJ, Mandelson MT. Colorectal cancer risk in relation to antidepressant medication use. Int J Cancer. 2011;128:227–32. 10.1002/ijc.25322. 20232382 10.1002/ijc.25322 PMC2962879

[24] Von Elm E, Altman DG, Egger M, Pocock SJ, Gøtzsche PC, Vandenbroucke JP. The Strengthening the Reporting of Observational Studies in Epidemiology (STROBE) statement: guidelines for reporting observational studies. Lancet. 2007;370:1453–7. 10.1016/S0140-6736(07)61602-X. 18064739 10.1016/S0140-6736(07)61602-X

[25] Weibull CE, Boman SE, Glimelius B, Syk I, Matthiessen P, Smedby KE, et al. CRCBaSe: a Swedish register-based resource for colorectal adenocarcinoma research. Acta Oncol. 2023;1–8. 10.1080/0284186X.2023.2197121. 10.1080/0284186X.2023.2197121 37029990

[26] WHO Collaborating Centre for Drug Statistics Methodology. ATC classification index with DDDs, 2025. [Internet]. 2025 [cited 2025 Apr 24]. https://atcddd.fhi.no/atc_ddd_index_and_guidelines/atc_ddd_index/. Accessed 24 Apr 2025.

[27] Salim A, Delcoigne B, Villaflores K, Koh W, Yuan J, Van Dam RM, et al. Comparisons of risk prediction methods using nested case-control data. Stat Med. 2017;36:455–65. 10.1002/sim.7143. 27734520 10.1002/sim.7143

[28] R Core Team. R: A Language and Environment for Statistical Computing [Internet]. Vienna, Austria: R Foundation for Statistical Computing; 2023. https://www.R-project.org/.

[29] Terry M, Therneau, Patricia M. Grambsch. Modeling Survival Data: Extending the Cox Model. New York: Springer; 2000.

[30] Wells L, Östberg V. How do educational disparities in smoking develop during early life? A Swedish longitudinal study. SSM - Popul Health. 2021;15:100859. 10.1016/j.ssmph.2021.100859. 34286059 10.1016/j.ssmph.2021.100859 PMC8274329

[31] Kobayashi LC, Wardle J, Von Wagner C. Limited health literacy is a barrier to colorectal cancer screening in England: Evidence from the English Longitudinal Study of Ageing. Prev Med. 2014;61:100–5. 10.1016/j.ypmed.2013.11.012. 24287122 10.1016/j.ypmed.2013.11.012 PMC3969575

[32] Svendsen MT, Bak CK, Sørensen K, Pelikan J, Riddersholm SJ, Skals RK, et al. Associations of health literacy with socioeconomic position, health risk behavior, and health status: a large national population-based survey among Danish adults. BMC Public Health. 2020;20:565. 10.1186/s12889-020-08498-8. 32345275 10.1186/s12889-020-08498-8 PMC7187482

[33] Bray F, Laversanne M, Sung H, Ferlay J, Siegel RL, Soerjomataram I, et al. Global cancer statistics 2022: GLOBOCAN estimates of incidence and mortality worldwide for 36 cancers in 185 countries. CA Cancer J Clin. 2024;74:229–63. 10.3322/caac.21834. 38572751 10.3322/caac.21834

[34] Offical Statistics of Sweden. Population Statistics [Internet]. 20250321. https://www.scb.se/en/finding-statistics/statistics-by-subject-area/population-and-living-conditions/population-composition-and-development/population-statistics/

[35] Coogan PF, Strom BL, Rosenberg L. Antidepressant use and colorectal cancer risk. Pharmacoepidemiol Drug Saf. 2009;18:1111–4. 10.1002/pds.1808. 19623565 10.1002/pds.1808 PMC2783290

[36] Cronin-Fenton DP, Riis AH, Lash TL, Dalton SO, Friis S, Robertson D, et al. Antidepressant use and colorectal cancer risk: a Danish population-based case–control study. Br J Cancer. 2011;104:188–92. 10.1038/sj.bjc.6605911. 20877356 10.1038/sj.bjc.6605911 PMC3039807

[37] Kiridly-Calderbank JF, Sturgeon SR, Kroenke CH, Reeves KW. Antidepressant Use and Risk of Colorectal Cancer in the Women’s Health Initiative. Cancer Epidemiol Biomarkers Prev. 2018;27:892–8. 10.1158/1055-9965.EPI-17-1035. 29789327 10.1158/1055-9965.EPI-17-1035 PMC6072592

[38] Heffner JL, Strawn JR, DelBello MP, Strakowski SM, Anthenelli RM. The co-occurrence of cigarette smoking and bipolar disorder: phenomenology and treatment considerations. Bipolar Disord. 2011;13:439–53. 10.1111/j.1399-5618.2011.00943.x. 22017214 10.1111/j.1399-5618.2011.00943.x PMC3729285

[39] Ziedonis D, Hitsman B, Beckham JC, Zvolensky M, Adler LE, Audrain-McGovern J, et al. Tobacco use and cessation in psychiatric disorders: National Institute of Mental Health report. Nicotine Tob Res. 2008;10:1691–715. 10.1080/14622200802443569. 19023823 10.1080/14622200802443569

[40] Kisely S, Seth R, Jordan SJ, Kendall B, Siskind DJ, Sara G, et al. Participation in the National Bowel Cancer Screening Program by people with severe mental illness, Australia, 2006–2019: a national data linkage study. Med J Aust. 2024;221:617–22. 10.5694/mja2.52521. 39537556 10.5694/mja2.52521 PMC11625528

[41] Hu H, Fu J, Han L, Gao G, Zhang W, Yu S, et al. The Antipsychotic Drug Aripiprazole Suppresses Colorectal Cancer by Targeting LAMP2a to Induce RNH1/miR-99a/mTOR‐Mediated Autophagy and Apoptosis. Adv Sci. 2024;11:2409498. 10.1002/advs.202409498. 10.1002/advs.202409498 PMC11672294 39513392

