# Supplementary material for: Risk of colorectal cancer by socioeconomic position and history of mental illness – a national nested case-control study
Source: BMC Cancer. 2026 Apr 25;26:527. doi: 10.1186/s12885-026-16093-0 (PMC13112772; doi:10.1186/s12885-026-16093-0)
Supplement: Supplementary file 1 — Supplementary Material 1. [file 12885_2026_16093_MOESM1_ESM.pdf]

SUPPLEMENTARY MATERIAL

Risk of colorectal cancer by socioeconomic position and history of mental illness – a national nested case-control study

Erik Osterman MD[1,2], Elisavet Syriopoulou [3], Anna Martling[1,4], Therese M-L Andersson [3], Caroline Nordenvall [1,4]
1 Department of Molecular Medicine and Surgery, Karolinska Institute, Sweden,
2 Department of Surgical Sciences, Uppsala University and Department of Surgery, Uppsala University Hospital, Sweden
3 Department of Medical Epidemiology and Biostatistics, Karolinska Institute, Sweden
4 Department of Pelvic Cancer, Colorectal Surgery Unit, Karolinska University Hospital, Sweden

Table of contents

Supplementary Figures 2
- Supplementary figure 1: Forest plot for the risk of Colorectal cancer by prescription for mental illness (MI) 2

Supplementary Tables 3
- Supplementary Table 1: Hazard ratio of colorectal cancer, colon and rectal cancer and colorectal cancer by sex obtained from models including all SEP indicators. 3
- Supplementary Table 2: Hazard ratio of colorectal cancer, colon and rectal cancer and colorectal cancer by sex obtained from a model including all SEP indicators as well as history of mental illness. 4
- Supplementary table 3: Prescriptions and specific psychiatric comorbidity of cases of colorectal cancer in Sweden 2008-2021 and controls from CRCBaSe. 5
- Supplementary table 4: Hazard ratio of colorectal cancer obtained from models adjusted with all SEP indicators, history of mental illness, as well as one prescribed drug category at a time. 6

Supplementary Figures

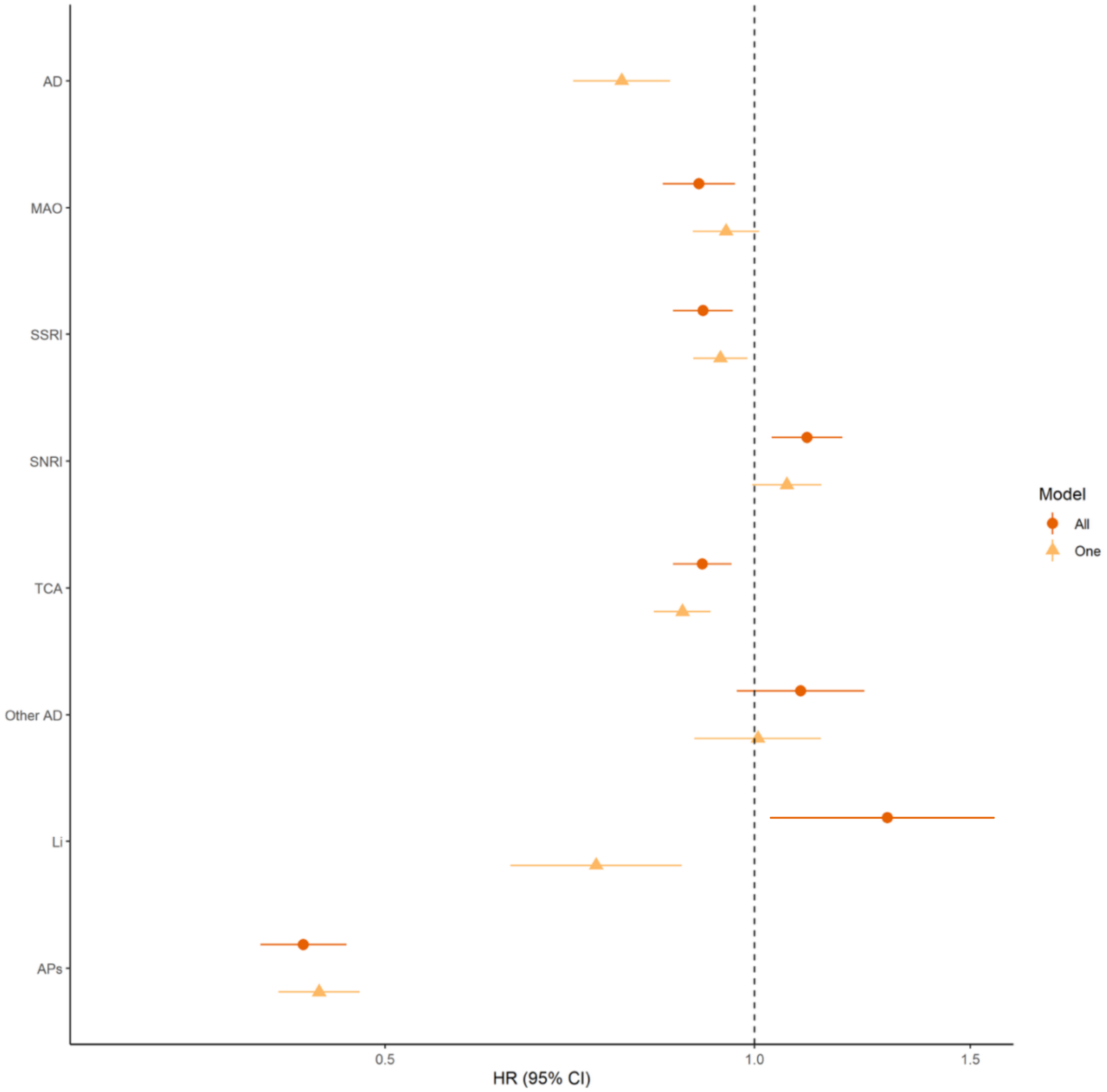

Supplementary figure 1: Forest plot for the risk of Colorectal cancer by prescription for mental illness (MI). Hazard Ratios were obtained from conditional logistic regression models with bars representing 95% confidence intervals. Matching by sex, age, and county of residence. Hazard ratios are obtained from a) models adjusted for history of mental illness, income, education, civil status, birth country as well as one prescription at a time (Model One in yellow, Table 4), and b) models where all prescriptions were entered simultaneously (Model All in orange , Table 3) with the same adjustments (all SEP indicators and history of mental illness). AD: All antidepressants, MAO: Drugs acting on MAO receptors or uptake pumps, SSRI: Selective Serotonin reuptake inhibitors, SNRI: Serotonin and Norepinephrine reuptake inhibitors, TCA: Atypical tetracyclic antidepressants, OtherAD: Other antidepressants, Li: Lithium, APs: Other antipsychotic medications

Supplementary Tables

Supplementary Table 1: Hazard ratio of colorectal cancer, colon and rectal cancer and colorectal cancer by sex obtained from models including all SEP indicators.

Exposure	Indicator Level	CRC *HR (95% CI)*	Colon *HR (95% CI)*	Rectal *HR (95% CI)*	Female *HR (95% CI)*	Male *HR (95% CI)*
**Income**	Q2 vs Q1	1.03 (1.01-1.06)	1.04 (1.01-1.06)	1.02 (0.98-1.06)	1.03 (0.99-1.06)	1.03 (1.00-1.07)
	Q3 vs Q1	1.02 (1.00-1.05)	1.02 (0.99-1.05)	1.01 (0.97-1.05)	1.02 (0.99-1.06)	1.02 (0.99-1.05)
	Q4 vs Q1	0.97 (0.94-0.99)	0.97 (0.94-1.00)	0.97 (0.93-1.01)	0.97 (0.94-1.01)	0.97 (0.93-1.00)
**Education**	9-12y vs <9y	1.02 (0.99-1.04)	1.03 (1.01-1.06)	0.99 (0.95-1.03)	1.02 (0.99-1.05)	1.01 (0.99-1.04)
	>12y vs <9y	0.98 (0.96-1.00)	1.01 (0.98-1.04)	0.92 (0.88-0.96)	0.98 (0.95-1.02)	0.98 (0.95-1.01)
**Birth country**	Nordic vs Sweden	1.04 (1.00-1.08)	1.04 (1.00-1.09)	1.02 (0.96-1.09)	1.00 (0.95-1.05)	1.08 (1.03-1.14)
	EU vs Sweden	1.09 (1.04-1.14)	1.07 (1.02-1.13)	1.12 (1.03-1.21)	1.00 (0.93-1.06)	1.17 (1.10-1.24)
	Non-EU vs Sweden	0.85 (0.81-0.88)	0.85 (0.81-0.90)	0.82 (0.77-0.88)	0.80 (0.75-0.85)	0.88 (0.83-0.92)
**Civil status**	Partner vs not	1.01 (0.99-1.02)	1.01 (0.99-1.03)	1.04 (1.01-1.07)	1.02 (0.99-1.04)	1.02 (0.99-1.04)

Sex, age, and county of residence matched controls. Conditional logistic regression.
All SEP: All SEP indicators entered simultaneously. E.g. adjusted for each
95%CI: 95% Confidence interval
EU: Non-Nordic EU countries
HR: Hazard ratio
Q: Quartile
SEP: Socioeconomic position
Y: Years of education

Supplementary Table 2: Hazard ratio of colorectal cancer, colon and rectal cancer and colorectal cancer by sex obtained from a model including all SEP indicators as well as history of mental illness.

Exposure	Indicator Level	CRC *HR (95% CI)*	Colon *HR (95% CI)*	Rectal *HR (95% CI)*	Female *HR (95% CI)*	Male *HR (95% CI)*
**Income**	Q2 vs Q1	1.04 (1.01-1.06)	1.04 (1.01-1.07)	1.02 (0.98-1.06)	1.03 (1.00-1.07)	1.04 (1.01-1.07)
	Q3 vs Q1	1.03 (1.00-1.05)	1.03 (1.00-1.06)	1.02 (0.97-1.06)	1.03 (0.99-1.06)	1.02 (0.99-1.06)
	Q4 vs Q1	0.97 (0.95-1.00)	0.97 (0.95-1.01)	0.97 (0.93-1.02)	0.98 (0.94-1.02)	0.97 (0.94-1.01)
**Education**	9-12y vs <9y	1.01 (0.99-1.03)	1.03 (1.00-1.06)	0.99 (0.95-1.02)	1.02 (0.99-1.05)	1.01 (0.99-1.04)
	>12y vs <9y	0.98 (0.96-1.00)	1.01 (0.98-1.04)	0.92 (0.88-0.96)	0.98 (0.95-1.01)	0.98 (0.95-1.01)
**Birth country**	Nordic vs Sweden	1.04 (1.00-1.08)	1.04 (1.00-1.09)	1.02 (0.96-1.09)	0.99 (0.95-1.04)	1.08 (1.02-1.14)
	EU vs Sweden	1.09 (1.04-1.14)	1.07 (1.02-1.13)	1.12 (1.03-1.20)	0.99 (0.93-1.06)	1.17 (1.10-1.24)
	Non-EU vs Sweden	0.85 (0.82-0.89)	0.86 (0.82-0.90)	0.82 (0.77-0.88)	0.80 (0.76-0.85)	0.88 (0.84-0.93)
**Civil status**	Partner vs not	1.01 (0.99-1.03)	1.01 (0.99-1.03)	1.04 (1.01-1.07)	1.02 (0.99-1.04)	1.02 (1.00-1.04)
**Mental illness**	Mild vs no	0.81 (0.78-0.83)	0.88 (0.85-0.91)	0.80 (0.76-0.83)	0.86 (0.83-0.88)	0.85 (0.82-0.88)
	Severe vs no	1.85 (1.74-1.96)	2.02 (1.89-2.16)	1.67 (1.50-1.86)	1.85 (1.70-2.01)	1.97 (1.82-2.13)

Sex, age, and county of residence matched controls. Conditional logistic regression.
Mental illness and SEP: History of mental illness and all SEP entered simultaneously.
95%CI: 95% Confidence interval
EU: Non-Nordic EU countries
HR: Hazard ratio
Q: Quartile
SEP: Socioeconomic position
Y: Years of education

Supplementary table 3: Prescriptions and specific psychiatric comorbidity of cases of colorectal cancer in Sweden 2008-2021 and controls from CRCBaSe.

**Characteristic**	**Total N (%)**	**Control N (%)**	**Case N (%)**
**Mental Illness group**			
None	441,125 (86.6%)	372,953 (86.5%)	68,172 (87.4%)
Mild depression	61,809 (12.1%)	53,533 (12.4%)	8,276 (10.6%)
Severe depression	92 (0.0%)	70 (0.0%)	22 (0.0%)
Psychosis	3,905 (0.8%)	3,225 (0.7%)	680 (0.9%)
Bipolar	2,217 (0.4%)	1,324 (0.3%)	893 (1.1%)
**MAO**			
No	498,444 (97.9%)	421,805 (97.8%)	76,639 (98.2%)
Yes	10,704 (2.1%)	9,300 (2.2%)	1,404 (1.8%)
**SSRI**			
No	463,342 (91.0%)	391,416 (90.8%)	71,926 (92.2%)
Yes	45,806 (9.0%)	39,689 (9.2%)	6,117 (7.8%)
**SNRI**			
No	500,149 (98.2%)	423,398 (98.2%)	76,751 (98.3%)
Yes	8,999 (1.8%)	7,707 (1.8%)	1,292 (1.7%)
**TCA**			
No	492,064 (96.6%)	416,162 (96.5%)	75,902 (97.3%)
Yes	17,084 (3.4%)	14,943 (3.5%)	2,141 (2.7%)
**Other antidepressive**			
No	506,710 (99.5%)	429,006 (99.5%)	77,704 (99.6%)
Yes	2,438 (0.5%)	2,099 (0.5%)	339 (0.4%)
**N05 (Antipsychotics)**			
No	498,627 (97.9%)	421,791 (97.8%)	76,836 (98.5%)
Yes	10,521 (2.1%)	9,314 (2.2%)	1,207 (1.5%)
**Lithium**			
No	507,878 (99.8%)	430,031 (99.8%)	77,847 (99.7%)
Yes	1,270 (0.2%)	1,074 (0.2%)	196 (0.3%)
**Antipsychotics (not Lithium)**			
No	498,650 (97.9%)	421,811 (97.8%)	76,839 (98.5%)
Yes	10,498 (2.1%)	9,294 (2.2%)	1,204 (1.5%)

MAO: Drugs acting on MAO receptors or uptake pumps, SSRI: Selective Serotonin reuptake inhibitors, SNRI: Serotonin and Norepinephrine reuptake inhibitors, TCA: Atypical tetracyclic antidepressants

Supplementary table 4: Hazard ratio of colorectal cancer obtained from models adjusted with all SEP indicators, history of mental illness, as well as one prescribed drug category at a time.

**Exposure**	**Indicator Level**	**Any antidepressant** *HR (95% CI)*	**MAO** *HR (95% CI)*	**SSRI** *HR (95% CI)*	**SNRI** *HR (95% CI)*	**TCA** *HR (95% CI)*	**Other antidepressant** *HR (95% CI)*	**Lithium** *HR (95% CI)*	**Antipsychotics** *HR (95% CI)*
**Medication**	Yes vs no	0.78 (0.71-0.85)	0.95 (0.89-1.01)	0.94 (0.89-0.99)	1.06 (1.00-1.13)	0.87 (0.83-0.92)	1.01 (0.89-1.13)	0.74 (0.63-0.87)	0.44 (0.41-0.48)
**Mental illness**	Mild vs no	1.02 (0.93-1.12)	0.81 (0.79-0.84)	0.84 (0.81-0.88)	0.80 (0.78-0.82)	0.83 (0.81-0.86)	0.81 (0.78-0.83)	0.81 (0.79-0.83)	0.86 (0.84-0.88)
	Severe vs no	1.91 (1.80-2.03)	1.85 (1.75-1.97)	1.86 (1.76-1.98)	1.85 (1.74-1.96)	1.86 (1.76-1.98)	1.85 (1.74-1.96)	1.90 (1.79-2.02)	2.92 (2.72-3.13)
**Income**	Q2 vs Q1	1.04 (1.02-1.06)	1.04 (1.02-1.06)	1.04 (1.02-1.06)	1.04 (1.02-1.06)	1.04 (1.02-1.06)	1.04 (1.02-1.06)	1.04 (1.02-1.06)	1.03 (1.01-1.06)
	Q3 vs Q1	1.03 (1.00-1.05)	1.03 (1.00-1.05)	1.03 (1.00-1.05)	1.03 (1.00-1.05)	1.03 (1.00-1.05)	1.03 (1.00-1.05)	1.03 (1.00-1.05)	1.02 (0.99-1.04)
	Q4 vs Q1	0.97 (0.95-1.00)	0.97 (0.95-1.00)	0.97 (0.95-1.00)	0.97 (0.95-1.00)	0.97 (0.95-1.00)	0.97 (0.95-1.00)	0.97 (0.95-1.00)	0.96 (0.94-0.99)
**Education**	9-12y vs <9y	1.02 (0.99-1.04)	1.02 (0.99-1.04)	1.02 (0.99-1.04)	1.02 (0.99-1.04)	1.02 (0.99-1.04)	1.02 (0.99-1.04)	1.02 (0.99-1.04)	1.01 (0.99-1.04)
	>12y vs <9y	0.98 (0.96-1.00)	0.98 (0.96-1.00)	0.98 (0.96-1.00)	0.98 (0.96-1.00)	0.98 (0.96-1.00)	0.98 (0.96-1.00)	0.98 (0.96-1.00)	0.98 (0.96-1.00)
**Birth country**	Nordic vs Sweden	1.04 (1.00-1.08)	1.04 (1.00-1.08)	1.04 (1.00-1.08)	1.04 (1.00-1.08)	1.04 (1.00-1.08)	1.04 (1.00-1.08)	1.04 (1.00-1.08)	1.04 (1.00-1.08)
	EU vs Sweden	1.09 (1.04-1.14)	1.09 (1.04-1.14)	1.09 (1.04-1.14)	1.09 (1.04-1.14)	1.09 (1.04-1.14)	1.09 (1.04-1.14)	1.09 (1.04-1.14)	1.09 (1.04-1.14)
	Non-EU vs Sweden	0.85 (0.82-0.89)	0.85 (0.82-0.89)	0.85 (0.82-0.89)	0.85 (0.82-0.89)	0.85 (0.82-0.89)	0.85 (0.82-0.89)	0.85 (0.82-0.89)	0.85 (0.82-0.89)
**Civil status**	Partner vs not	1.01 (0.99-1.02)	1.01 (0.99-1.02)	1.01 (0.99-1.02)	1.01 (0.99-1.02)	1.01 (0.99-1.02)	1.01 (0.99-1.02)	1.01 (0.99-1.02)	1.00 (0.98-1.02)

Each column represents HRs from one model. Each model contained one medication, mental illness and SEP.
MAO: Drugs acting on MAO receptors or uptake pumps, SSRI: Selective Serotonin reuptake inhibitors, SNRI: Serotonin and Norepinephrine reuptake inhibitors, TCA: atypical tetracyclic antidepressants, Other antidepressants, Lithium, Other antipsychotic medications
